# A content analysis of popular media reporting regarding increases in minimum ages of legal access for tobacco

**DOI:** 10.1186/s12889-018-6020-6

**Published:** 2018-09-17

**Authors:** Jocelyn Huey, Dorie E. Apollonio

**Affiliations:** Department of Clinical Pharmacy, University of California, 3333 California Street, Suite 420, San Francisco, CA 94143-0613 USA

## Abstract

**Background:**

In the late 20th century, US localities began increasing the minimum age of legal access (MLA) for tobacco from 18 to 21 years by enacting “Tobacco 21” ordinances. Although these policies have a strong evidence base and broad popular support, popular media coverage of tobacco control laws has not always been accurate. This study sought to determine if contemporaneous popular media reporting accurately reflected the scientific findings regarding increased tobacco MLAs.

**Methods:**

We searched LexisNexis for popular media reports that (1) addressed proposed or enacted Tobacco 21 ordinances and were (2) published in English, (3) drawn from a US news source, and (4) written after January 2004. We conducted a content analysis for quality based on a validated measure of accuracy of reporting, the Index of Scientific Quality (ISQ), which allows assessment of articles by assigning scores ranging from 1 (lowest) to 5 (highest).

**Results:**

Searches yielded 378 articles; after screening for relevance and duplicates, 98 were included in the review. All studies identified through the keyword searches addressed Tobacco 21 policies. The average global score identifying the scientific quality of the articles was 2.98 of 5. Over three-quarters of the popular media articles addressing Tobacco 21 laws were written after a systematic review of these policies was released by the Institute of Medicine and approximately 4 in 10 cited findings from that review.

**Conclusions:**

Popular media reports on Tobacco 21 laws demonstrated average overall quality and relied on both anecdotal and scientific evidence, in contrast to previous studies found that popular media reports on tobacco issues demonstrated low overall quality and relied primarily on anecdotal evidence. The systematic review of increased MLAs for tobacco written by the Institute of Medicine diffused quickly into popular reporting, suggesting that this type of evidence might improve research translation.

## Background

Tobacco use is the leading preventable cause of death in United States and the negative health consequences of tobacco use have been well established for decades [1]. In 2015, approximately 4.7 million middle and high school students in the US were current tobacco smokers [2]. Nine out of ten of smokers begin smoking before age 18, and smoking behavior among young adults is predictive of smoking in later years [1, 3, 4]. Despite evidence of tobacco industry marketing toward youth and young adults, [5] policies to reduce access to tobacco for this group have been limited in scope [2].

In the late 20th century, localities in the United States instituted renewed efforts to increase the MLA for tobacco from 18 to 21 years, generally referred to as “Tobacco 21” laws [6]. These efforts resulted in a nearly 50% decrease in cigarette smoking rates among high-school students (13% to 7%) and a comparable decrease in store purchases of cigarettes (18% to 13%) [7]. In 2015 the Institute of Medicine projected that increasing the MLA for tobacco to 21 years would reduce adult smoking by 12% and prevent 223,000 premature deaths [4]. Tobacco 21 policies are popular: 70% of adults support raising the MLA for tobacco to 21 years, including a majority of adults in all demographic and smoking status categories [8]. However despite clinical evidence and popular support, as of 2016 only two states (California and Hawaii) had increased their MLA for tobacco to 21 years; as of 2017, an additional three states (Maine, New Jersey, and Oregon) had done so [6].

Existing studies of research translation detail the process from the generation of research to its use by policymakers [9, 10]. These studies have identified the importance of systematic reviews in translating evidence into policy, [11–14] and note that dissemination strategies that involve contact with policymakers are critical [15–18] because most policymakers are not trained to interpret scientific research or rewarded for doing so [19, 20]. The nature of reporting affects public opinion, influences individual behavior, and plays a central role in the process of public health policy formation [21, 22]. Although partisanship, ideology, and maintaining consistent voting records all factor into policymakers’ decisions, the extent of public support for proposed policies offers critical information in making decisions about whether to enact such changes [23].

Media misunderstanding of research findings is common [24]. Multiple studies report that journalists translate research evidence poorly, particularly during novel events [25–27]. Past studies suggest that the accuracy of research translation by journalists covering tobacco issues has been inconsistent [28, 29]. The limited research on the scientific accuracy of popular reporting on tobacco has led to calls for additional research in this area [30–33].

To address this gap, we sought to assess the accuracy of popular media reporting on Tobacco 21 laws. The coverage of proposed increases in MLAs for tobacco offers particular insight in understanding research translation because it addresses two issues anticipated to affect the accuracy of popular media reports: novelty and systematic reviews. Tobacco 21 policies became relevant over a limited time period; the issue first became relevant in the 21st century after the passage of a Tobacco 21 ordinance in Needham, Massachusetts in 2005 [7]. In March 2015, the US Institute of Medicine (IOM) published a systematic review of the effects of increasing MLAs for tobacco [4]. Following the publication of this report Hawaii passed a Tobacco 21 law in June 2015, and California passed a similar law in June 2016 [6]. Consistent with existing research, we hypothesized that (1) popular reporting on Tobacco 21 laws would rely heavily on anecdotal evidence; and (2) the publication of the IOM report would lead to higher quality popular media reports.

## Methods

We conducted a content analysis of popular news articles that addressed increased MLAs for tobacco. We focused on articles in the public domain that were most likely to be easily found by individuals who were inexperienced with traditional academic research methods. To identify these reports, one of the authors (JH) searched the LexisNexis database for newspaper and magazine articles with the assistance of a university librarian. The search was conducted in May 2016 and relied on relevant keywords: “smoking” AND “tobacco” AND (“smoking age” OR “legal age” OR “minimum age” OR ((“teen age” OR “adolescent”) AND “tobacco control”) or “tobacco 21”). We included articles from newspapers and newswires that were (1) published in English, (2) drawn from a US news source, and (3) written after January 2004. This start date was chosen because it was one year prior to the first local US implementation of a Tobacco 21 policy in the 21st century. We excluded duplicate articles and articles that assessed smoking cessation and other clean air policies.

The following information was extracted from each article or website by one reviewer (JH):

- Title of the article
- Publication type (e.g., newspaper, magazine article, wire service stories)
- Publication date

We relied on a validated instrument created by Oxman et al., [34] the Index of Scientific Quality (ISQ), to assess the quality of popular media reports. The ISQ index uses a five-point scale, with 1 corresponding to the lowest level of quality and 5 corresponding to the highest level of quality. A score of 4 or 5 indicates clear references to evidence, while a score of 2 or 3 represents partly or definitely unclear references to evidence. An ISQ score of 1 is assigned to criteria where the evidence base is potentially misleading. We modified the ISQ coding instrument to reflect outcomes relevant to Tobacco 21 laws. The applicability measure restricted the topic specifically to MLAs for tobacco; the validity measure relied both on specific terms (e.g. “prestigious” used as a marker for quality) and mention of systematic reviews; the magnitude measure included measures of health outcomes related to tobacco; the consequences considered health outcomes specific to tobacco such as smoking rates and costs. Details regarding the coding of each content area are provided in the Appendix. In addition, the instrument was expanded so that both coders made a judgment regarding whether the article, taken overall, claimed that increasing the tobacco sales age to 21 was effective, ineffective, or took no position. We used the IOM report as a gold standard for assessing reporting of relevant research.

The instrument covered the following content areas:

1. Applicability: Describes whether or not the author clearly refers to the affected population (21 and under)
2. Opinions versus Facts: Describes whether or not facts are clearly distinguished from opinions
3. Validity: Describes whether or not the assessment of the credibility (validity) of the evidence is clear and well-founded (not misleading)
4. Magnitude: Describes whether or not the strength or magnitude of the findings (effects on smoking rate, health, or costs) that are the main focus of the article are clearly reported
5. Precision: Describes whether or not the author provides a clear and well-founded (not misleading) assessment of the precision of any estimates that are reported or of the probability that any of the reported findings might be due to chance
6. Consistency: Describes whether or not the consistency of the evidence (between studies) is considered and whether the assessment is well-founded (not misleading)
7. Consequences: Describes whether or not all of the important consequences (youth and adult smoking rates, deaths from tobacco use, health care costs, sales and government revenue) of concern relative to the central topic of the report are identified
8. Global: Describes the overall scientific quality of the report

The analysis of article quality relied on the mean quality scores in each category identified by the ISQ, with subgroup analyses conducted for articles published before and after the release of the IOM report. In coding for content, both authors reviewed each article using the instrument, working independently. Cohen’s κ was run to assess interrater reliability for each ISQ quality criteria. κ was interpreted by the guidelines from Altman (1991) in which a κ score of 0.00–0.20 indicates poor agreement, 0.21–0.40 indicates fair agreement, 0.41–0.60 indicates moderate agreement, 0.61–0.80 indicates good agreement, and 0.81–1.00 indicates very good agreement [35]. Agreement was good for applicability and consistency; moderate for consequences and global, fair for opinions versus facts, validity, and precision, and poor for magnitude. Coding discrepancies in all categories were discussed and resolved by consensus.

## Results

**Fig. 1:**
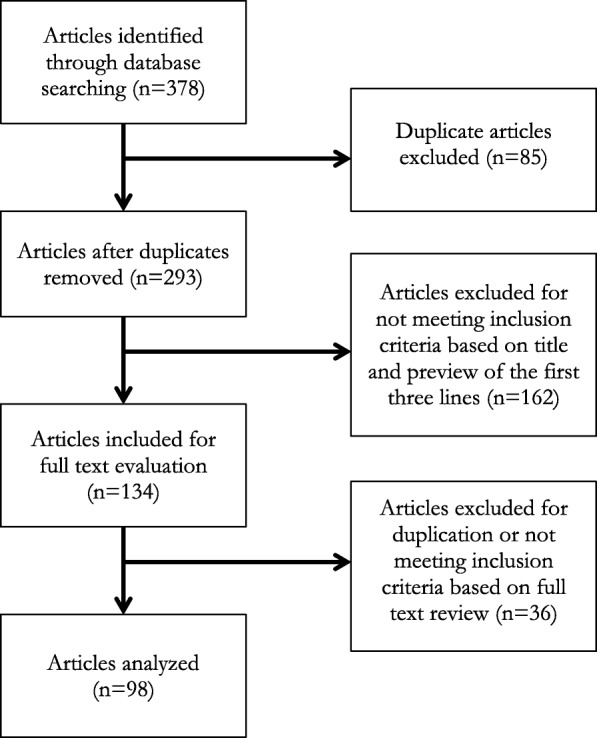
Flow of included articles regarding Tobacco 21 laws

The initial database searches identified 378 popular media articles. One of the authors (JH) screened these articles for relevance. Eighty-five articles were identified as duplicates based on title, word count, and preview of the first three lines, and were excluded from the analysis. An additional 162 articles were removed from analysis because they did not meet the inclusion criteria based on title and preview of the first three lines. The remaining 134 articles were eligible for full-text review by both authors. After reading these articles in full, an additional 36 were identified to be either duplicates or to not meet the inclusion criteria by consensus of both authors, leaving 98 articles included in the final analysis. The screening process is outlined in Fig. 1.

### Article characteristics

Publication dates ranged from 2006 to 2016. Eighty percent of the articles did not take a position on Tobacco 21 laws; of the remaining articles, 16% supported the policies and 4% opposed them, as shown in Table 1. The majority of articles (82%) were published after 2015, with only 18 articles published before 2015. An increase in reporting on Tobacco 21 was correlated with the introduction of Hawaii Senate Bill 1030 in January 2015, which first proposed to raise the state’s MLA for tobacco to 21 years.

**Table 1:** Tobacco 21 article characteristics (2004–2016), *n* = 98

*Characteristics*	*# Articles/Percentage*
Position	
--Neutral	78 (80%)
--Support	16 (16%)
--Oppose	4 (4%)
Time periods	
--Pre-Hawaii bill (2004 to January 27, 2015)	18 (18%)
--Post-Hawaii bill introduction (January 28, 2015 and forward)	80 (82%)
--Pre-IOM report (2015)	22 (22%)
--Post-IOM report	76 (78%)

### Quality scores by content area

Table 2 provides the mean values and SDs for each of the ISQ quality criteria.

**Table 2:** Summary statistics for article quality

*Criteria*	*Overall mean ± SD*	*Pre-IOM report*	*Post-IOM report*
Applicability	5.00 ± 0.00	5.00 ± 0.00	5.00 ± 0.00
Opinions v. facts	3.92 ± 1.03	4.02 ± 1.10	3.91 ± 1.02
Validity	2.83 ± 0.49	2.84 ± 0.47	2.83 ± 0.50
Magnitude	3.84 ± 0.94	3.41 ± 0.89	3.52 ± 0.95
Precision	1.08 ± 0.45	1.14 ± 0.64	1.07 ± 0.38
Consistency	2.79 ± 1.71	1.95 ± 0.97*	3.04 ± 1.18*
Consequences	2.26 ± 1.14	2.05 ± 1.14	2.34 ± 1.13
Global	2.98 ± 0.95	3.16 ± 0.98	2.94 ± 0.94

Criteria quality scores ranged from 1 (lowest) to 5 (highest); *p < 0.001

#### Applicability

Each article clearly stated that it considered Tobacco 21 policies so applicability received an average score of 5, the highest ranking for all criteria. Although the search strategy was designed to identify articles addressing Tobacco 21 laws, previous research has found that popular media articles do not always accurately reference policies that are putatively being reported or assessed.

#### Opinion v. facts

Distinction of opinion v. facts averaged 3.92, the second highest ranking for all criteria, indicating that articles were more evidence-based than opinion-based. Of the total 98 articles, 35 (36%) received a 5, indicating all factual claims were quoted or cited, and two received a 1, which meant opinions were offered as facts without qualification.

#### Validity

Validity represented the journalist’s assessment of the quality of evidence used the article. A score of 1 indicates that research was misrepresented, 2 that research was not referenced, 3 that studies were presented without discussion of their quality, 4 that the article made unqualified claims, and 5 that there was some discussion about why a study was “good” such as a reference to the weight of evidence. The articles scored an average of 2.83 for validity, a score representing average quality.

#### Magnitude

The magnitude of findings, which referred to the extent to which claims about effects were anchored with data averaged 3.84, the third highest quality ranking across criteria, suggesting that the articles made both general and specific claims about the potential effects of a Tobacco 21 policy. A score of 1 indicated that effects either were not mentioned or were misrepresented, 2 that effects were implied but not explicitly mentioned, 3 that effects were discussed in general terms, 4 that exact figures assessing outcomes were mixed in with general claims, and 5 that the article relied on exact percentages or estimates of the numbers of lives saved.

#### Precision

Assessment of the precision of results due to study design scored an average of 1.08, the lowest ranking for all criteria; a score of 1 indicated there was no indication of whether results were due to chance, 3 that there was some effort to link study design to credibility, and 5 that there was an explanation of study design.

#### Consistency

Consistency of evidence between studies, referencing the number of studies discussed and the accuracy with which they represented the state of contemporaneous research, scored 2.79 for all articles, suggesting average quality. Articles that did not cite a specific study or that used a potentially misleading source of data were assigned a score of 1, while discussions of one, two, or three or more studies were scored 2, 3, and 4 respectively. Articles that referred to a systematic review, such as the IOM report, were scored 5.

#### Consequences

We tallied the number of consequences relevant to Tobacco 21 that were mentioned in articles, specifically potential effects on smoking rates, deaths from tobacco use, health care costs, and sales of tobacco and/or tax receipts. On average, 2.26 consequences were listed, with 12 articles listing 4 or more potential effects of the policy and 19 articles listing either one potential effect or none.

#### Overall quality

The average global score identifying the scientific quality of the articles was 2.98 of a potential 5, representing average quality. Misleading articles scored were scored as 1, those that treated evidence equally with opinion scored 2, those that included some opinion but had more weight on evidence scored 3, those that presented claims that were evidence focused but not explained scored 4, and articles in which major claims were supported by evidence and explained scored 5.

#### Before and after the IOM report

The 2015 report by the Institute of Medicine found that increasing the MLA for tobacco products would prevent or delay use of such products by adolescents, improve population health, and reduce tobacco-related deaths. Table 2 also provides a comparison of the mean values and SDs prior to and after the IOM report. Our review found that 76 (78%) articles were written after the report was released; of these, 43% cited findings from the IOM’s report. After the release of the IOM report the quality scores for consistency, which represented the number of studies discussed and their representation of current research, improved from 1.95 to 3.04; this difference was statistically significant (*p* < 0.001). Scores also increased for magnitude and consequences but the differences were not statistically significant. Scores decreased for opinions v. facts, validity, and precision; these differences were also not statistically significant.

### Nature of arguments

Proponents and opponents of Tobacco 21 policies included in the articles used different types of arguments, with proponents focused on outcomes and opponents focused on ideological claims, as shown in Table 3.

**Table 3:** Nature of arguments in Tobacco 21 articles (2004–2016)

*Types of arguments*	*# Articles/Percentage*
Proponents	
Reduces youth smoking rate	78 (80%)
Reduces deaths	50 (51%)
Reduces adult smoking rate	42 (43%)
Reduces healthcare costs	34 (35%)
Changes tax revenue	16 (16%)
Public support	7 (7%)
Opponents	
Individual decision making paramount	37 (38%)
Attested link between age of military service and MLA	32 (33%)
Negative financial impact	15 (15%)
Individuals will circumvent the law	15 (15%)

#### Proponents

Supporters of Tobacco 21 policies primarily referenced scientific studies that focused on the prevalence of smoking and the health consequences of increasing the MLA. Among the five major consequences analyzed, the impact on youth smoking rate (80% of articles) and deaths due to smoking (51%) were the most frequently mentioned effects. Articles also referred to effects on adult smoking rate (43%), health care cost (35%), and revenue (16%), however these issues were discussed less frequently. Approximately 7% of articles cited statistics that demonstrated strong public support for the policies, particularly among current or former smokers. Supportive claims were typically made by public health professionals or legislators speaking on tobacco-related issues, rather than the general public.

#### Opponents

Consistent with past arguments against stronger tobacco control policies, the concerns expressed by opponents of Tobacco 21 policies primarily focused on the individual rights to make decisions rather than on research findings regarding the effects of the policies or on tobacco industry marketing to youth. About 38% of articles claimed that Tobacco 21 laws would impede individual decision making; opponents argued that increasing the MLA was tantamount to creating a “nanny state” that interfered with the decisions of young adults. These claims often focused on extended analogy; 33% of articles stated that if people were old enough to vote and enlist in the military, they were old enough to smoke. In 15% of articles, opponents of the policies speculated that despite research showing that Tobacco 21 policies had resulted in reduced tobacco use, young people might circumvent the law by purchasing tobacco in neighboring jurisdictions with lower MLAs or obtain tobacco from family and friends. In 15% of the articles, critics of the policies attempted to shift focus from the potential of an increased MLA to save lives and reduce health care costs by making counterclaims that such policies would have a negative financial impact on small businesses and government by reducing tobacco sales and tobacco tax revenue.

## Discussion

This study provides the first assessment of popular media coverage addressing laws that increased tobacco MLAs to 21 years. Consistent with previous studies, we hypothesized that popular reporting would demonstrate low overall quality and rely on anecdotal evidence. Instead, we found that media reports on this topic were of average quality and relied on both anecdotal and scientific evidence. Our content analysis found that applicability, opinion v. facts, and magnitude were the highest scoring categories, indicating that articles were focused on Tobacco 21 policies and mostly reported facts and figures assessing their effects. The views of public health advocates were better represented than those of the tobacco industry. However, when reporting on claims made by opponents to the policies, articles disproportionately relied on their anecdotes and speculation, rather than research findings. We found that measures of precision were consistently weak, suggesting that the concept of statistical significance and the role of chance remains difficult to communicate through popular media reports. This finding is consistent with previous research; one study suggested that reporters preferentially cover medical research with weaker methodology [27].

We also hypothesized that publication of the IOM report would lead to higher quality popular media reports. The scores for consistency showed a statistically significant increase in quality, suggesting that journalists recognize the value of systematic reviews over individual studies. These findings also appeared in claims made by policy advocates; in contrast, policy opponents lacked comparable evidence and relied on ideological or anecdotal claims. Our results are consistent with previous research that attempted to train consumer advocates to better understand and communicate research. The majority of advocates believed that systematic reviews were more reliable than individual studies after training, even though less than half stated that they were comfortable with analyzing research methods and designs [36]. The differences in scores for other measured categories of scientific quality were not statistically significant.

This research has limitations. We may not have identified all published articles in LexisNexis, given that our inclusion criteria limited our selection to articles published from January 2004 to March 2016. Data collection stopped shortly after the passage of California law, making it possible that later articles were missed. Coverage may increase again if additional states propose and enact Tobacco 21 laws. In addition, we focused on written media, and did not assess reporting in television, radio, or social media. Finally, our findings with respect to Tobacco 21 laws may not be generalizable to other aspects of tobacco control.

## Conclusions

Our findings provide new evidence about translation of clinical research into community settings, and help fill a gap in understanding the accuracy of media reports on tobacco issues. Consistent with the continued concern about the quality of popular media reporting on scientific research, we found that reporting on Tobacco 21 policies was of average quality and inconsistently cited data from scientific studies. Our results also show that while a systematic review addressing this topic diffused relatively quickly into popular reporting, it was not always referenced. Nonetheless these findings suggest that systematic reviews appear to improve popular media reporting with respect to communicating the overall state of research evidence. Development of policy-relevant systematic reviews may be a useful strategy to help reduce tobacco-related disease by communicating information about research evidence to policymakers and the public.

## Appendix

**Table 4:** Coding instrument

Applicability	1	Refers to unrelated age limits
	3	Mixes discussion of 21 and other age limits
	5	Clearly refers to population (21 and under)
Opinions v. facts	1	Opinions offered as facts without qualification
	3	Mix of citations and opinions offered as facts
	5	All factual claims either quoted or cited
Validity	1	Research misrepresented
	2	No reference to research
	3	Refers to study(ies) but no further discussion
	4	Makes unqualified claim of validity “prestigious” or “irrefutable”
	5	Some discussion of why study(ies) good (systematic review, weight of evidence)
Magnitude	1	No mention of effects or effects misrepresented
	2	Effects implied but not explicitly mentioned
	3	Refers to “reduction” or “increase” without specifics
	4	Mixes exact figures with general claims
	5	Exact percentages or lives saved estimates
Precision	1	No indication of whether results are due to chance
	3	Some effort to link study design to credibility of results
	5	Discusses alternative explanations, sampling, or omitted variable bias, etc.
Consistency	1	Potentially misleading selection of studies (e.g. “studies show”)
	2	One study discussed
	3	Two studies discussed
	4	Three or more studies discussed
	5	Reference to body of evidence or to a systematic review (IOM report)
Consequences	0	No reference to consequences
(Count #)	1	Affects youth smoking rate
	2	Affects adult smoking rate
	3	Affects deaths from tobacco use
	4	Affects health care costs
	5	Affects sales or government revenue (e.g. taxes lost from reduced sales)
Global	1	Misleading
	2	Evidence treated equally with opinion
	3	Some opinion included but weight of article is on evidence
	4	Evidence is focus of article but not explained
	5	Major claims supported by evidence and explained
Writer’s conclusion		Supports
		Opposes
		No opinion given

## Abbreviations

IOM: Institute of Medicine
ISQ: Index of Scientific Quality
MLA: Minimal age of legal access
